# Influence of Oxygen–Plasma Treatment on In-Situ SiN/AlGaN/GaN MOSHEMT with PECVD SiO_2_ Gate Insulator

**DOI:** 10.3390/ma12233968

**Published:** 2019-11-29

**Authors:** Geunho Cho, Ho-young Cha, Hyungtak Kim

**Affiliations:** School of Electronic and Electrical Engineering, Hongik University, Seoul 04066, Korea; 1220cho@hanmail.net (G.C.); hcha@hongik.ac.kr (H.-y.C.)

**Keywords:** gallium nitride, heterostructure, in situ SiN, SiO_2_, oxygen plasma, interface trap

## Abstract

The influence of oxygen–plasma treatment on in situ SiN/AlGaN/GaN MOS high electron mobility transistor with SiO_2_ gate insulator was investigated. Oxygen–plasma treatment was performed on in situ SiN, before SiO_2_ gate insulator was deposited by plasma-enhanced chemical vapor deposition (PECVD). DC I-V characteristics were not changed by oxygen plasma treatment. However, pulsed I-V characteristics were improved, showing less dispersion compared to non-treated devices. During short-term gate bias stress, the threshold voltage shift was also smaller in a treated device than in an untreated one. X-ray photoemission spectroscopy also revealed that SiO_2_ on in situ SiN with oxygen–plasma treatment has an O/Si ratio close to the theoretical value. This suggests that the oxygen plasma treatment-modified surface condition of the SiN layer is favorable to SiO_2_ formation by PECVD.

## 1. Introduction

AlGaN/GaN heterojunction-based metal insulator semiconductor high electron mobility transistors (MISHEMTs) have demonstrated excellent performance for high-efficiency power switching applications [1,2,3]. Although one can employ a gate dielectric in MISHEMTs to suppress gate leakage current and magnify gate swing, the bulk properties of the gate dielectric and the dielectric/III-nitride interface quality strongly influence the device performance because this dielectric layer also plays an important role in alleviating the current slump caused by the surface trapping. However, high-density interface trap states present at the dielectric/AlGaN barrier interface are in the order of ~10^13^–10^14^ cm^−2^eV^−1^ [4,5,6]. Although ex-situ deposition, such as low pressure CVD and atomic layer deposition, is a common method for gate dielectrics of GaN transistors, an in situ SiN dielectric can be epitaxially grown immediately as a final layer of the AlGaN/GaN HEMT epi-structure in a metal–organic chemical vapor deposition (MOCVD) system [7,8,9]. Without the exposure of the as-grown GaN HEMT barrier to the ambient air, the damage or contamination caused by subsequent processing can be minimized. Therefore, in situ SiN passivation or a protection layer has recently been widely accepted in a commercial GaN-on-Si wafer. 

Among dielectric materials, SiO_2_ is a very attractive material for MIS structure, since it possesses a large band gap and large conduction band discontinuity from AlGaN. Chemical vapor deposition of SiO_2_ on AlGaN/GaN heterostructure has been reported by several research teams, and AlGaN/GaN MOSHEMT demonstrated a promising performance [10,11,12]. As mentioned above, an in situ SiN layer has been employed as standard surface passivation for commercially available AlGaN/GaN heterostructure substrate. SiN can be etched away prior to SiO_2_ deposition for MOSEMT device fabrication. However, plasma-based dry-etching, which is prone to induce plasma damage on the surface, is required, because in situ SiN cannot be removed easily by wet etching. If a SiO_2_ layer can be deposited on in situ SiN with the dielectric quality maintained, we can gain all the advantages of employing in situ SiN passivation and a SiO_2_ gate insulator. Although SiO_2_ can be thermally grown on SiN, the process takes place at very high temperatures and causes damage to the material. 

In this work, we carried out oxygen plasma treatment on in situ SiN on AlGaN/GaN-on-Si substrate before SiO_2_ deposition. The effect of oxygen plasma treatment on the characteristics of the SiO_2_ layer deposited on in situ SiN was investigated by measuring MOSHEMT’s electrical characteristics, including I-V, C-V and gate lag measurements. Short-term gate stress test and X-ray photoemission spectroscopy (XPS) were also performed to analyze the characteristics of the SiO_2_/in situ SiN/AlGaN MOS structure.

## 2. Materials and Methods

**Figure 1:**
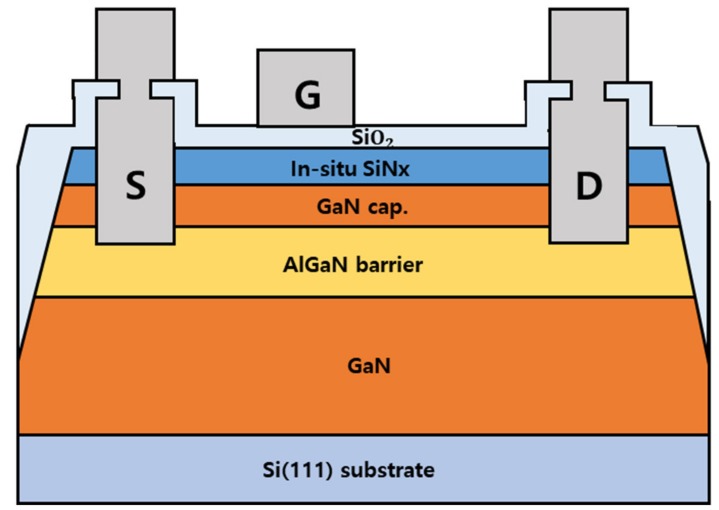
Cross-sectional diagram of in situ SiN/AlGaN/GaN metal–organic semiconductor high electron mobility transistor (MOS-HEMT) on Si (not scaled).

As presented in Figure 1, AlGaN/GaN MOSHEMTs were fabricated on commercially available in situ SiN/AlGaN/GaN heterostructures grown on 6 in silicon substrates by MOCVD. The epilayers, from bottom to top, consist of a 4.5 μm GaN buffer, a 490 nm unintentionally doped GaN channel layer, a 22 nm Al0.25Ga0.75N barrier, a 4 nm GaN cap, and a 10 nm in situ SiN cap. The layer structure information was provided by the wafer provider, Enkris Semiconductor Inc. in Suzhou, China. The Hall mobility (μ) and sheet carrier concentration (n_sh_) of the 2DEG channel at heterojunction was 1890 cm^2^/V·s and 8.85 × 10^12^, respectively. Circular-type MOS capacitors were also fabricated on the same sample. The device fabrication was done at Inter-university Semiconductor Research Center in Seoul National University, Korea. It started with a recess etching of the AlGaN barrier through in situ SiN to reduce a contact resistance [recess]. A Ti/Al/Ni/Au metal stack (20/120/25/50 nm) was deposited by e-beam evaporation for source/drain electrodes. The ohmic contacts were alloyed by rapid thermal annealing at 830 ℃ for 30 s in N_2_. Device isolation was performed using mesa formation with a depth of 300 nm. The surface was submitted to oxygen plasma treatment using a microwave asher with 50 W power and 40 sccm flow rate for 2 min, before SiO_2_ deposition. Following that, a 20 nm PECVD SiO_2_ layer was deposited for gate insulation and device passivation. Finally, an Ni/Au-based metal stack (20/200 nm) was deposited to form the gate and probe pad. The average ohmic contact resistance was determined to be 0.85 Ω-mm, and the sheet resistance of 2-DEG was ~470 Ω/sq, as measured by the transfer length method (TLM) after the fabrication was complete. Gate length and width of the MOSHEMT were 2 and 100 μm, respectively.

## 3. Results

**Figure 2:**
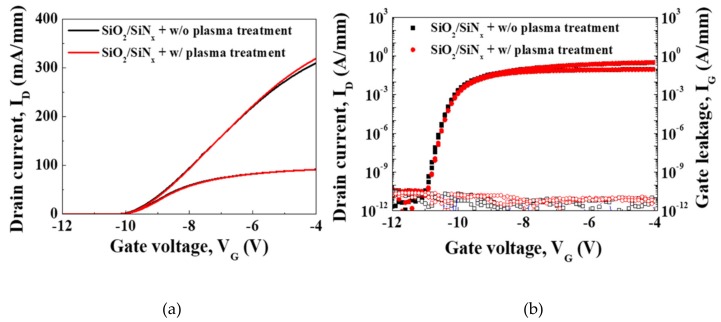
(**a**) Transfer I-V characteristics measured at V_D_ = 1 and 10 V. (**b**) Subthreshold (solid mark) and gate leakage (open mark) characteristics measured at V_D_ = 1 and 10 V.

**Figure 3:**
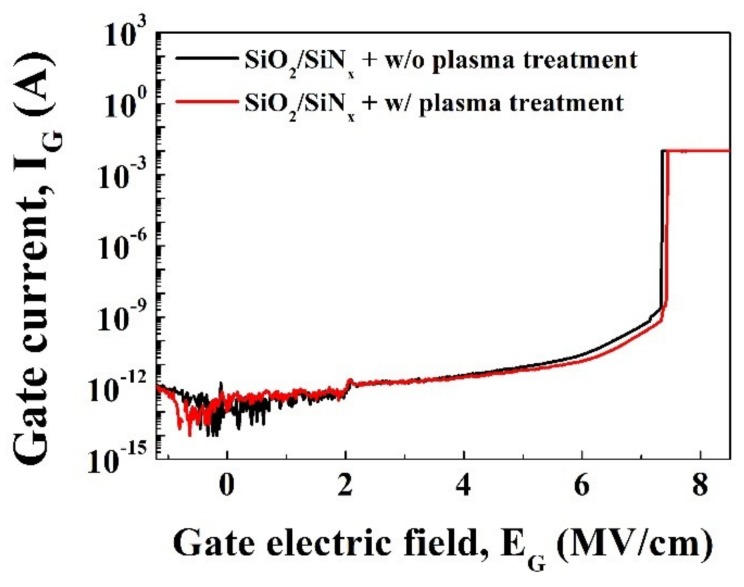
Time-zero-breakdown characteristics of SiO_2_/in situ SiN/AlGaN/GaN MOS structure.

Transfer I-V characteristics of the fabricated devices were measured at room temperature by Agilent 4155 semiconductor parameter analyzer. As shown in Figure 2a, oxygen plasma treatment did not change device characteristics including on-current, a threshold voltage (V_th_) and transconductance. Figure 2b shows that neither gate leakage current nor subthreshold swing was degraded by plasma treatment. Time-zero-breakdown characteristics were also evaluated to compare the integrity of the gate oxide and did not show significant differences between the two samples, as given in Figure 3.

**Figure 4:**
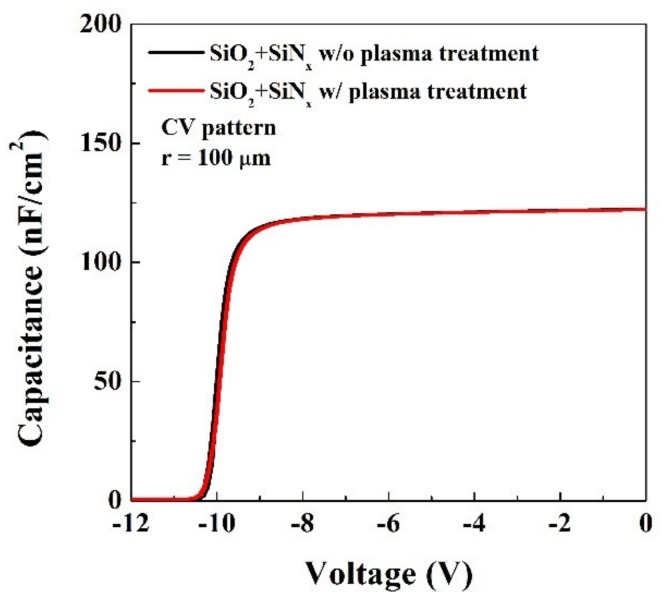
C-V characteristics of SiO_2_/in situ SiN/AlGaN/GaN MOS capacitor measured at 1 MHz.

C-V characteristics were measured at f = 1 MHz from a circular-shaped C-V pattern with radius = 100 um using Agilent B1500A. Figure 4 shows negligible differences in C-V characteristics between two types of devices with or without plasma treatment. This suggests that the dielectric thickness and constant were not altered by the plasma treatment.

**Figure 5:**
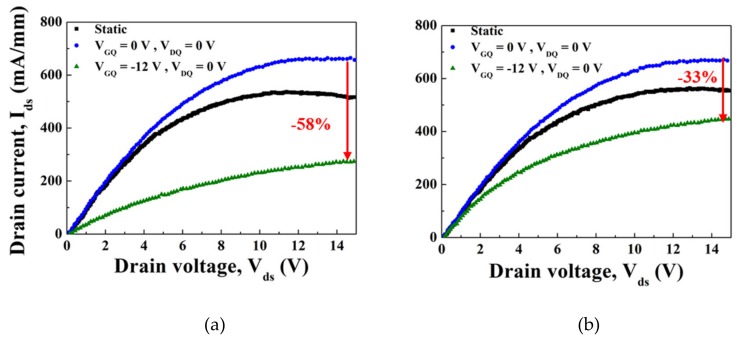
Pulsed I-V (gate lag) characteristics measured with 1 us-VG pulsed width in a 0.1 % duty cycle. (**a**) Without plasma treatment (**b**) With plasma treatment.

Pulsed I-V measurement is a useful tool in analyzing gate lag or drain lag phenomenon caused by the trapping effect [13]. Gate lag characteristics have a strong correlation with RF output performance, which can be improved significantly by minimizing gate lag, i.e., the dispersion between DC and pulsed I-V characteristics [14]. The virtual gate effect, proposed by Vetury et al. [15], is well-known as an origin of the dispersion. Gate lag measurement was performed to investigate the effect of plasma treatment on the trapping phenomenon observed in GaN-based HEMTs by using the Accent DiVA D265 Dynamic analyzer. Quiescent bias points for the two pulsed measurements were V_G_ = V_D_ = 0 V and V_G_ = −12, V_D_ = 0 V. The pulse width was 1 μs with 1 ms of the pulse period. V_GQ_ = −12 V was chosen to ensure that the DUT was completely pinched off and electrons were easily injected from the gate into the traps nearby. Figure 5 shows the pulsed and static I-V characteristics and the difference between static and pulsed I-V. (V_G_ = V_D_ = 0 V) resulted from the self-heating effect. As shown in Figure 5, the MOSHEMT without oxygen plasma treatment exhibited a larger I–V dispersion between the two measurement conditions. Although 38% dispersion was observed in the plasma-treated device, the untreated device showed 58% dispersion at V_D_ = 15 V. This result suggests a more serious virtual gate effect [15] and the ineffectiveness of the suppression of current collapse in the devices without plasma treatment.

## 4. Discussion

When AlGaN/GaN MOS-HEMTs were treated by oxygen plasma before SiO_2_ dielectric deposition, they did not show any changes in DC I-V and C-V characteristics, such as pinch-off voltage, transconductance and C_max_, indicating 2DEG property was not altered. Channel and Gate leakage characteristics were not degraded, either.

**Figure 6:**
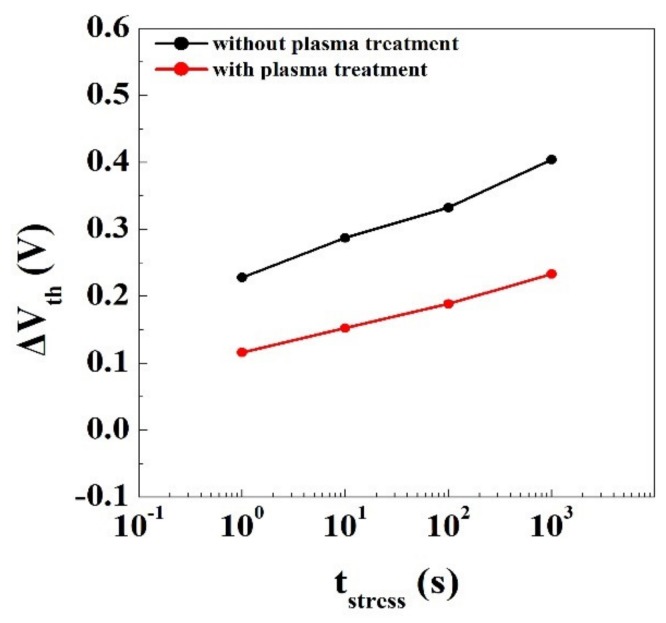
ΔV_th_ induced by a short-term gate bias stress measured from the plasma-treated and non-treated device.

However, pulsed I-V measurement revealed that gate lag was aggravated if the surface of in situ SiN was not exposed to oxygen plasma before SiO_2_ deposition. To investigate how the trap states of a SiO_2_/SiN/AlGaN MOS structure can be modified by oxygen plasma treatment, the devices were submitted to a short-term stress test and ΔV_th_ was monitored. The ΔV_th_ value can easily be translated into an interface trap density using the oxide capacitance [16]. In Figure 6, a plasma-treated device showed ΔV_th_ less than that of an untreated one during the bias stress, with the same electric field and time, suggesting that the interface trap density was decreased by plasma treatment.

**Figure 7:**
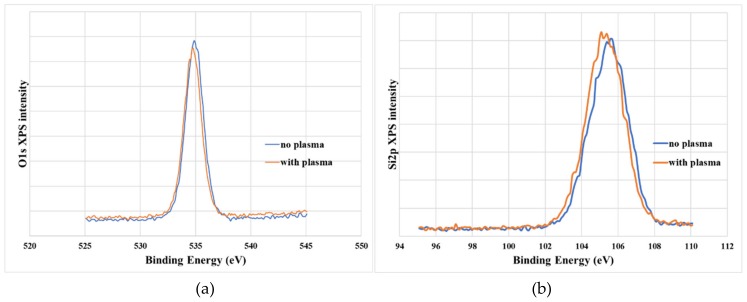
XPS spectrum of O1s and Si2p in the vicinity of the SiO2/SiN interface. Sputter etching for 150 s. (**a**) O1s (**b**) Si2p.

**Figure 8:**
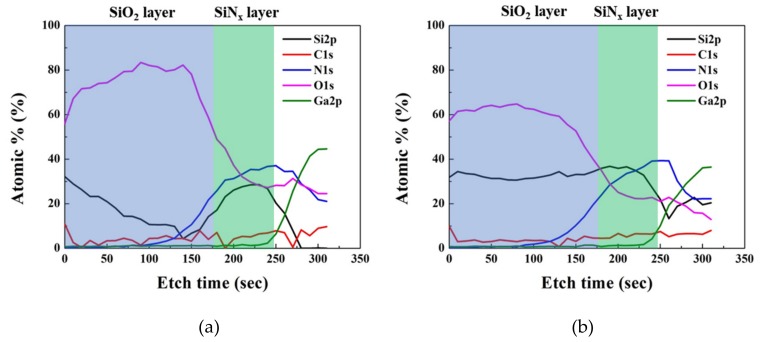
Atomic percent ratio vs. sputter time SiO_2_/in situ SiN/AlGaN MOS structure. (**a**) Without plasma treatment (**b**) With plasma treatment.

We carried out further analysis of the SiO_2_/SiN/AlGaN MOS structure to understand the origin of the reduced trapping observed from the oxygen–plasma treated device. To quantify the oxygen composition in the SiO_2_ film, x-ray photoelectron spectroscopy (XPS) measurements with sputter etch treatment was performed with the thetaprobe XPS system from Thermo Fisher Scientific at Analytical Instrumentation Center of Hanyang University, Seoul, Korea. A monochromatic Al Κα X-ray source with 1486.6 eV and sputtering ion gun with Ar+ at 2 keV were used for analysis. At the top surface with no sputter etching, Si 2p and O 1s peak positions (not shown in Figure) are determined to be 103.08 and 532.58 eV for the untreated sample, respectively. Only a little change in binding energy was observed in the plasma-treated sample, showing each peak at 103.48 and 532.98 eV. Figure 7 also presents Si 2p and O 1s peaks, with 150 s of sputter etch time which is suggested to give a depth near the interface between ex-situ SiO_2_ and in situ SiN. No significant changes in the XPS spectrum were observed. However, the XPS depth profile of each element in Figure 8 reveals that SiO_2_ on in-situ SiN with oxygen–plasma treatment has an O/Si ratio close to the theoretical value (2:1). This suggests that oxygen–plasma treatment modified the surface of in-situ SiN in favor of the following SiO_2_ deposition. Plasma oxidation of PECVD and LPCVD nitride has been already demonstrated to produce oxynitride films [17,18] Oxygen–plasma was also applied to convert the surface of silicon nitride into oxide [19]. Buiu et al. proposed that this conversion was due to a loss of nitrogen into the plasma caused by the bonding instability of nitrogen at the surface of the dielectric [20]. Several reports proposed similar mechanisms that weakly bind nitrogen at the surface of silicon nitride can be replaced by oxygen [19,21]. The oxidized surface of silicon nitride by oxygen plasma can probably provide better conditions for ex-situ deposition of SiO_2_.

The current work is aimed at optimizing the treatment process with varying power and oxygen flow to further improve the SiO_2_/SiN interface property. A more detailed characterization of the plasma-treated surface before SiO_2_ deposition is now in progress. Besides, the effects of plasma treatment on the chemical bonding and morphology of the dielectrics will accompany this.

## 5. Conclusions

We investigate the effect of oxygen–plasma treatment on in-situ SiN on AlGaN/GaN heterojunction for MOS gate devices. Oxygen–plasma treatment was performed before a SiO_2_ gate insulator was deposited on in situ SiN by PECVD. Oxygen–plasma treatment did not change DC I-V characteristics. However, the dispersion between DC and pulsed I-V characteristics was significantly alleviated by the plasma treatment. Accordingly, the plasma-treated device exhibited a smaller Vth shift than the untreated one, suggesting that the trapping into the interface states was mitigated. The XPS result revealed that the SiO_2_ layer on the plasma-treated sample exhibited an O/Si ratio much closer to the theoretical value than the untreated one. In cases where a SiO_2_ layer is employed as a gate insulator on an AlGaN/GaN-on-Si platform with an in situ SiN capping layer, oxygen plasma treatment on the in situ SiN layer can improve the quality of SiO_2_ significantly.
